# *Echinococcus granulosus* Infection Results in an Increase in *Eisenbergiella* and *Parabacteroides* Genera in the Gut of Mice

**DOI:** 10.3389/fmicb.2018.02890

**Published:** 2018-11-29

**Authors:** Jianling Bao, Huajun Zheng, Yuezhu Wang, Xueting Zheng, Li He, Wenjing Qi, Tian Wang, Baoping Guo, Gang Guo, Zhaoxia Zhang, Wenbao Zhang, Jun Li, Donald P. McManus

**Affiliations:** ^1^State Key Laboratory of Pathogenesis, Prevention and Treatment of High Incidence Diseases in Central Asian, The First Affiliated Hospital of Xinjiang Medical University, Urumqi, China; ^2^College of Public Health, Xinjiang Medical University, Urumqi, China; ^3^Key Laboratory of Reproduction Regulation of NPFPC, SIPPR, IRD, Fudan University, Shanghai, China; ^4^Shanghai-MOST Key Laboratory of Health and Disease Genomics, Chinese National Human Genome Center at Shanghai, Shanghai, China; ^5^Molecular Parasitology Laboratory, QIMR Berghofer Medical Research Institute, Brisbane, QLD, Australia

**Keywords:** *Echinococcus granulosus*, cystic echinococcosis, mice, microbiome, immunoglobins

## Abstract

Cystic echinococcosis (CE) is a chronic infectious disease caused by *Echinococcus granulosus*. To confirm whether the infection impacts on the gut microbiota, we established a mouse model of *E. granulosus* infection in this study whereby BALB/c mice were infected with micro-cysts of *E. granulosus*. After 4 months of infection, fecal samples were collected for high-throughput sequencing of the hypervariable regions of the 16S rRNA gene. Sequence analysis revealed a total of 13,353 operational taxonomic units (OTUs) with only 40.6% of the OTUs having genera reference information and 101 of the OTUs were significantly increased in infected mice. Bioinformatics analysis showed that the common core microbiota were not significantly changed at family level. However, two genera (*Eisenbergiella* and *Parabacteroides*) were enriched in the infected mice (*P*_AMOV A_ < 0.05) at genus level. Functional analysis indicated that seven pathways were altered in the *E. granulosus* Infection Group compared with the Uninfected Group. Spearman correlation analysis showed strong correlations of IgG, IgG1 and IgG2a with nine major genera. *E. granulosus* cyst infection may change the gut microbiota which may be associated with metabolic pathways.

## Introduction

Cystic echinococcosis (CE) is a cosmopolitan zoonosis caused by the cystic stage of the dog tapeworm *Echinococcus granulosus* (McManus et al., 2012). The disease causes serious health problems and economic losses, especially in Central Asia (including western China), northern Africa and South America (McManus et al., 2012). *E. granulosus* requires two hosts [an intermediate host including sheep, goats, cattle or wild herbivores and a definitive host such as dogs (or wolves and other carnivores)] to complete its life-cycle. Humans also become infected as an incidental host by ingesting eggs released from *E. granulosus* in carnivore feces. After hatching in the stomach and intestine, the eggs (oncospheres) penetrate the gut mucosa, enter the blood circulation and finally develop in internal organs mainly the liver (70%) and lungs (20%) of humans. Serological studies showed that in endemic areas, 4–26% of the population were seropositive against *E. granulosus* antigens (Gavidia et al., 2008), indicating that a large number of individuals had been infected. CE infection strongly impacts on host immune responses (Zhang et al., 2003, 2012) with high levels of antibodies, particularly IgG1, IgG4 and IgE (Zhang et al., 2003) and predominant Th2 cytokines including IL-4, IL-5, IL-6, and IL-10 (Rigano et al., 1997; Casaravilla et al., 2014), indicating that the host immune response against CE differs from a bacterial or viral infection (Zeng et al., 2017).

The gut microbiota play an important role in human health (Chen et al., 2017) impacting on metabolism, immunity, development and the behavior of the host (Thaiss et al., 2016). In addition, microbiota components are impacted by medical conditions such as cancer (Jensen et al., 2015; O’Keefe, 2016; Sonnenburg and Backhed, 2016). Similar changes occur in experimental models as well (Gkouskou et al., 2014; Yu et al., 2018). Studies showed that helminth infection in the gut induced typical Th2 immune responses which may control the mcriobiota in the gut of mice (Ramanan et al., 2016; Guernier et al., 2017; Wegener Parfrey et al., 2017). However, it is not known whether *E. granulosus* infection impacts on the gut microbiota of humans or mice. Mice have been used for *E. granulosus* larval infection including primary (Zhang et al., 2001) and secondary infection (Gottstein, 2001; Mourglia-Ettlin et al., 2016). Mice models play an important role in studies of developmental biology and host specificity in echinococcosis (Nakaya et al., 2006). Recently, mouse models were successfully used for drug screening and development (Elissondo et al., 2007; Wang et al., 2017). To increase the success of secondary infection, we developed a method using micro-cysts cultured *in vitro* to infect mice (Zhang et al., 2005), and obtained more than 70% of cyst recovery from 50 PSC-generated cysts.

In this study, BALB/C mice were infected with micro-cysts of *E. granulosus* and their fecal samples were collected for sequencing the variable regions of 16S rRNA genes of gut commensal bacteria to determine their composition and diversity. We show that *E. granulosus* impacted on the gut microbiota of the mice with microbiota changes likely being associated with the altered host immune status in infected individuals.

## Materials and Methods

### Ethics Statement

The protocols for using mice in the study were approved by the Ethics Committee of The First Affiliated Hospital of Xinjiang Medical University (FAH-XMU, Approval No. IACUC-20120625003). The “Guidelines for the Care of Laboratory Animals” by the Ministry of Science and Technology of the People’s Republic of China (2006) were rigidly followed in the use of these animals.

### Preparation of Cultured *E. granulosus* Hydatid Cysts

Fresh *E. granulosus* sensu stricto protoscoleces (PSC) were aspirated from hydatid cysts from sheep livers collected from a slaughterhouse in Urumqi, Xinjiang Uyghur Autonomous Region, China. The PSC were digested with 1% (w/v) pepsin and cultured to obtain echinococcal cysts using published procedures (Zhang et al., 2005; Wang et al., 2014).

### Animal Infection and Sample Collection

Pathogen-free female BALB/c mice, aged 6 weeks, were purchased from Beijing Vital River Laboratory Animal Technology Co., Ltd. All animals were housed at the animal facility of the FAH-XMU. The BALB/c mice were randomly divided into two groups: *E. granulosus* infected group (Infected Group) and uninfected group (Uninfected Group). The mice in the *E. granulosus* Infection Group were intraperitoneally (i.p) transplanted with 35 small (diameter, 200–300 μm) hydatid cysts suspended in 0.4 mL RPMI 1640 medium through a 1.0 mL syringe as described in our previous study (Zhang et al., 2005; Wang et al., 2016). Every mouse in the Uninfected Group was i.p. injected with 1.0 mL RPMI 1640 medium. After 4 months, the stool of each mouse was collected daily and about 1 g stool was collected over 1 week. The stool samples were stored at -80°C until use.

### Antibody Isotype and Subtype Assays

Mice were sacrificed after the last fecal sample collection, then serum samples were obtained from peripheral blood and stored at -20°C for use. The sera were analyzed by ELISA for different immunoglobulins to hydatid cyst fluid (HCF) including IgG, IgG1, IgG2a, IgG2b and IgG3 and IgM. In brief, each well of MaxiSorb immune-plates (Nunc International) was coated with 100 μL of sheep HCF antigens at a concentration of 2 mg/mL in carbonate/bicarbonate buffer (pH 9.6) (Kittelberger et al., 2002) and incubated overnight at 4°C. After three washes with PBS, the wells were each blocked with 200 μL of 5% (v/v) skim milk in PBS for 1 h at 37°C. Mouse serum was diluted 1–200. For each well, 100 μL of the diluted serum was added and incubated at 37°C for 1 h. After three washes with PBS containing 0.05% Tween 20 (PBST), each well was added 100 μL of diluted anti-mouse monoclonal IgG, IgG1, IgG2a, IgG2b and IgG3 and IgM conjugate (BETHYL). The reaction was developed by adding 100 μL of 2,2-azino-di-[ethyl-benzothiazoline sulfonate] substrate solution (Sigma). After incubation for 30 min in the dark at room temperature, optical density values were read at 405 nm by an ELISA reader (Thermo, Waltham, United States).

### Echinococcal Cyst Number and Size

The abdominal cavity of each mouse was opened after sacrifice, and hydatid cysts in the cavity were removed and the numbers of hydatid cysts were counted and their sizes were measured.

### PCR Amplification and 16S rRNA Gene Sequencing

Genomic DNA was extracted from stool samples using KAPA HiFi HotStart ReadyMix Kits (Kapa Biosciences, Woburn, MA, United States). The V3–V4 hypervariable region of the 16S rRNA gene was amplified by PCR and sequenced; the length of the V3–V4 hypervariable region was approximately 469 bp. Amplicon pools were prepared for sequencing with AMPure XT beads (Beckman Coulter Genomics, Danvers, MA, United States) and quantification with the Library Quantification Kit for Illumina (Kapa Biosciences, Woburn, MA, United States), respectively. The libraries were sequenced on 300PE MiSeq runs.

### Bioinformatics and Statistical Analysis

Mothur (version 1.39.5) was used to assemble the paired FASTQ files (Schloss et al., 2011). The selected quality DNA sequences were confirmed using the following criteria: (1) no contaminant sequences, (2) containing no ambiguous bases, (3) the size length ≥350 bp, (4) containing no chimeric sequences, and (5) primers were trimmed. The average length of selected DNA sequences was 414 bp (350–446 bp). The selected DNA sequences were then grouped into operational taxonomic units (OTUs) by comparing with SILVA reference databases (V128) (Quast et al., 2013) at 97% similarity. The minimum reads number of samples (24,097) was used for data normalization. Community richness, evenness and diversity analysis (Shannon, Simpson, Shannoneven, Simpsonenven, ACE, Chao and Good’s coverage) were analyzed using the Mothur *T*-test (with 95% confidence intervals, *p*-value <0.05). Taxonomy was assigned using the online software Ribosomal Database Project (RDP) classifier (80% threshold) (Wang et al., 2007) based on the RDP (Cole et al., 2009). LEfSe (Segata et al., 2011) was also performed to detect abundance taxa (*p*-value <0.05) between the two groups and estimate linear discriminant analysis effect size (LDA score >2.0). Then Mothur was performed to check the LEfSe results using “metastats” command. Differences among the two groups were also assessed using Analysis of Molecular Variance (AMOVA) in Mothur. Microbiome functions were analyzed using PICRUSt (Langille et al., 2013) based on the KEGG pathways by normalizing the 16S rRNA copy numbers. The input file (biom file) of PICRUSt was calculated using the Mothur software command “classify.otu” and “make.biom”, and then the input file was uploaded to the online PICRUSt for function analysis. Differences were determined using STAMP (Parks et al., 2014).

### Correlation of Antibody Isotypes and Bacteria

Statistical analysis program-R Package was performed to calculate the coefficient relationship between bacterial genera present and immunoglobulin isotypes using the non-parametric Spearman rank correlation algorithm. A coefficient of >0.68 or <-0.68 was considered to represent strong correlation (Taylor, 1990).

## Results

### Infection and Blood Serum Isotypes

**FIGURE 1:**
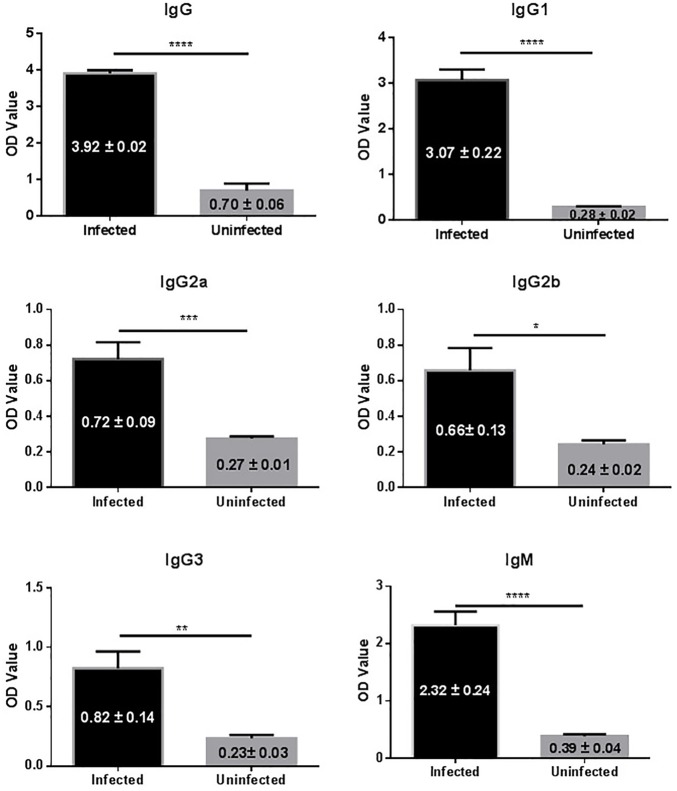
IgG antibody isotypes and subtypes in sera of mice infected with *E. granulosus*. ^∗^*p* < 0.05, ^∗∗^*p* < 0.01, ^∗∗∗^*p* < 0.005, ^∗∗∗∗^*p* < 0.001.

In this study 14 mice were transplanted with 35 micro-cysts of *E. granulosus*. All the mice were successfully infected with an average number of 16 (SD ± 7.0) cysts and an average size of 6.4 mm (0.1–21 mm) in diameter (Table 1). Serological antibody tests showed that these infected mice had a predominantly IgG1 antibody response against HCF antigens, followed by IgG2b, IgG2a and IgG3 (Figure 1), indicating *E. granulosus* infection induced a predominant Th2 response.

**Table 1:** The number and average size of cysts in the *E. granulosus* infection group.

	Eg1	Eg2	Eg3	Eg4	Eg5	Eg11	Eg12	Eg13	Eg14	Eg15	Eg31	Eg32	Eg33	Eg35
Number of cysts	12	15	17	37	21	16	17	14	15	11	12	8	23	13
Average size of cysts(cm)	0.58	0.54	0.58	0.72	0.74	0.55	0.67	0.42	0.49	0.71	0.55	0.75	1.01	0.77


*Eg, mice infected with *E. granulosus*.*

### Bacterial Populations in the Stool Samples

Stool samples from the 25 mice were collected for gastrointestinal microbiota analysis, including 14 samples from the mice infected with cysts of *E. granulosus* (Infected Group) and 11 control samples from mice without infection (Uninfected Group,). A total of 1,383,569 16S rRNA genes were identified by high-throughput DNA sequencing analysis after filtering through quality control filters. The gene numbers ranged from 24,097 (from one in Uninfected Group) to 86,478 genes. To normalize the data to avoid statistical bias, 24,097 genes from the mice with the lowest gene number were used as a baseline for normalization of all the sequences. OTU (97% similarity) analysis was used to estimate richness, evenness and diversity of the bacterial communities. A total of 13,353 OTUs were obtained including 9,118 OTUs from mice infected with *E. granulosus*, and 8,423 OTUs from the uninfected mice (Table 2). No significant difference was evident between the two groups of mice in term of OTU numbers (*p* > 0.05). The Good’s coverage was over 93.5% (93.5∼97.8%) for each sample, and over 98% for the two groups, respectively (Table 2), meaning that the sequencing depth was sufficient to undertake microbiota analysis with two groups.

**Table 2:** The diversity evaluation of the microbiomes of mice infected with cystic echinococcosis and uninfected mice.

				Richness		Evenness		Diversity	
Group	Sample	OTUs	Coverage (%)	Chao	ACE	Simpsoneven	Shannoneven	Shannon	Simpson
Infected	14	9,118	98.50	18,212.718	26,873.315	0.01444	0.646977	5.899138	0.007595
Uninfected	11	8,423	98.25	16,846.368	25,099.202	0.01675	0.660714	5.972012	0.007084


*Infected, mice infected with *E. granulosus*; Uninfected, uninfected mice; OTUs, operational taxonomic units; Chao, Chao index, ACE, ACE index.*

### Core Microbiome in the Gut of Mice

Ribosomal Database Project analysis showed that 99.7% of the 16S rRNA genes were aligned into nine phyla with the common bacteria *Firmicutes*, *Bacteroidetes* and *Proteobacteria* being dominant in both infected and uninfected groups. RDP analysis clustered 93.5% of the genes (OTUs) into 58 families and 13 families were identified as the major taxa and core microbiomes co-existing in the two groups. The genes in those families accounted for 91.61 and 94.27% of the microbiome community in the infected group and uninfected group, respectively (Table 3). Among the 13 families, *Lachnospiraceae* was mostly predominant in both groups, accounting for 41.42 and 43.92% of the total microbiome, respectively. *Ruminococcaceae* and *Porphyromonadaceae* were also dominant (>10% of the entire microbiome in both groups).

**Table 3:** The major families of microbiota in *E. granulosus* infected mice and uninfected mice.

Family	Uninfected (%)	Infected (%)	*p*-value
*Lachnospiraceae*	41.42	43.92	*p* > 0.05
*Porphyromonadaceae*	14.21	15.75	*p* > 0.05
*Ruminococcaceae*	11.52	10.87	*p* > 0.05
*Rikenellaceae*	6.61	5.33	*p* > 0.05
*Bacteroidaceae*	5.80	5.93	*p* > 0.05
*Helicobacteraceae*	3.31	3.94	*p* > 0.05
*Prevotellaceae*	2.32	1.94	*p* > 0.05
*Desulfovibrionaceae*	1.94	2.30	*p* > 0.05
*Coriobacteriaceae*	0.14	0.20	*p* > 0.05
*Deferribacteraceae*	1.82	2.73	*p* > 0.05
*Lactobacillaceae*	1.80	0.90	*p* > 0.05
*Enterobacteriaceae*	0.39	0.17	*p* > 0.05
*Erysipelotrichaceae*	0.33	0.29	*p* > 0.05


*Uninfected, uninfected mice; Infected, mice infected with *E. granulosus*.*

**FIGURE 2:**
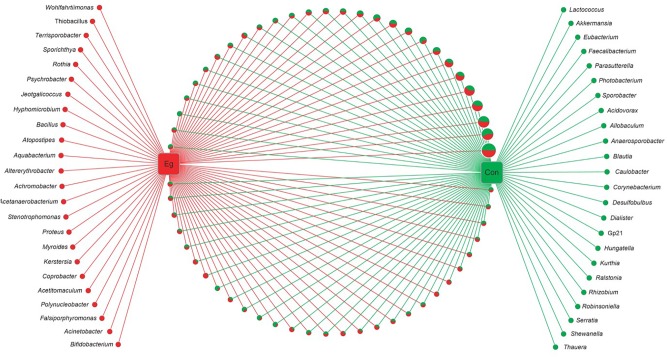
Networks of all bacterial genera revealed in *E. granulosus* uninfected and infected animal groups. Green circle: the unique genera of the Uninfected Group; red circle: the unique genera of Infected Group. In pie charts, green and red represents the genus proportion of the uninfected and infected animal groups; all the pie charts present all the genera common in both groups. Circle sizes represent the read numbers.

Among the 58 families, 40.4% of genes (OUTs) have genus reference information and were aligned into 105 classified genera (Figure 2). There were 57 genera co-existing in both groups. However, there were 24 genera present in the Infection Group and another 24 genera in the Uninfected Group. The proportion of all the group unique genera was less than 0.01%, and no significant differences were found between the two groups.

Among the 105 classified genera, 33 were core genera (with each genus comprising >0.1% of total the microbiome), including *Bacteroides*, *Odoribacter*, *Clostridium XlVa*, *Helicobacter*, *Alistipes*, *Barnesiella* and *Mucispirillum* (Table 4). Among the predominant genera, there were 27 ubiquitous (core) genera which were consistently found in all samples and comprised more than 38% of the total microbiome.

**Table 4:** The abundance of genera (>0.1%) in the gut microbiomes of *E. granulosus* infected mice and uninfected mice determined by LEfSe analysis.

Order	Family	Genus	Feature	Uninfected (%)	Infected (%)	*p*-value
Coriobacteriales	Coriobacteriaceae	Enterorhabdus	Ubiquitous	0.06	0.08	
Bacteroidales	Bacteroidaceae	Bacteroides	Ubiquitous	5.80	5.93	
Bacteroidales	Porphyromonadaceae	Barnesiella	Ubiquitous	2.06	2.99	
Bacteroidales	Porphyromonadaceae	Odoribacter	Ubiquitous	3.86	4.33	
Bacteroidales	Porphyromonadaceae	Parabacteroides	Ubiquitous	0.29^∗^	0.56^∗^	0.023976
Bacteroidales	Prevotellaceae	Alloprevotella	Ubiquitous	1.55	1.13	
Bacteroidales	Prevotellaceae	Prevotella	Ubiquitous	0.73	0.80	
Bacteroidales	Rikenellaceae	Rikenella	Ubiquitous	1.81	1.67	
Deferribacterales	Deferribacteraceae	Mucispirillum	Ubiquitous	1.81	2.72	
Lactobacillales	Lactobacillaceae	Lactobacillus	Ubiquitous	1.80	0.90	
Clostridiales	Clostridiaceae 1	Clostridium sensu stricto		0.12	0.17	
Clostridiales	Lachnospiraceae	Acetatifactor	Ubiquitous	1.10	1.01	
Clostridiales	Lachnospiraceae	Clostridium XlVa	Ubiquitous	2.70	4.19	
Clostridiales	Lachnospiraceae	Clostridium XlVb	Ubiquitous	0.40	0.28	
Clostridiales	Lachnospiraceae	Dorea	Ubiquitous	0.10	0.07	
Clostridiales	Lachnospiraceae	Eisenbergiella		0.02^∗^	0.35^∗^	0.001998
Clostridiales	Lachnospiraceae	Lachnospiraceae incertae_sedis	Ubiquitous	0.56	0.61	
Clostridiales	Lachnospiraceae	Roseburia	Ubiquitous	0.06	0.11	
Clostridiales	Ruminococcaceae	Anaerotruncus	Ubiquitous	0.31	0.31	
Clostridiales	Ruminococcaceae	Butyricicoccus	Ubiquitous	0.35	0.35	
Clostridiales	Ruminococcaceae	Clostridium IV	Ubiquitous	0.04	0.05	
Clostridiales	Ruminococcaceae	Flavonifractor	Ubiquitous	1.62	1.78	
Clostridiales	Ruminococcaceae	Intestinimonas	Ubiquitous	0.15^∗^	0.11^∗^	0.035964
Clostridiales	Ruminococcaceae	Oscillibacter	Ubiquitous	1.69	1.29	
Clostridiales	Ruminococcaceae	Pseudoflavonifractor	Ubiquitous	0.68	0.73	
Clostridiales	Ruminococcaceae	Ruminococcus		0.13	0.02	
Erysipelotrichales	Erysipelotrichaceae	Erysipelotrichaceae incertae_sedis	Ubiquitous	0.21	0.14	
Erysipelotrichales	Erysipelotrichaceae	Turicibacter		0.03	0.10	
Desulfovibrionales	Desulfovibrionaceae	Bilophila		0.03	0.03	
Desulfovibrionales	Desulfovibrionaceae	Desulfovibrio	Ubiquitous	0.34	0.67	
Campylobacterales	Helicobacteraceae	Helicobacter	Ubiquitous	3.31	3.94	
Enterobacteriales	Enterobacteriaceae	Escherichia/Shigella	Ubiquitous	0.39	0.17	
Anaeroplasmatales	Anaeroplasmataceae	Anaeroplasma		0.16	0.10	


*Uninfected, uninfected mice; Infected, mice infected with *E. granulosus*. ^∗^significantly different between uninfected mice and infected mice; ubiquitous, the genus was identified as present in all samples.*

At the OTU level, there were significant differences between the two groups; 101 OTUs were significantly increased and 49 OTU were significantly decreased in the infected mouse group (*p* < 0.05) (Table 5). Of note, most (59.6%) OTUs were unclassified into genera as no classification information is available for these OTUs.

**Table 5:** The abundance of OTU (*p* < 0.01) in the infected mice and uninfected mice determined by LEfSe analysis.

#OTU	Taxonomy	Uninfected: mean rel.freq.(%)	Infected: mean rel.freq.(%)	*p*-values
OTU00452	Clostridiales;Lachnospiraceae;Clostridium XlVa	0.0139587	0.04387	0.000351
OTU00048	Desulfovibrionales;Desulfovibrionaceae;unclassified_Desulfovibrionaceae	0.0067907	0.934319	0.000472
OTU00409	Clostridiales;Lachnospiraceae;Acetatifactor	0.0033954	0.040906	0.001051
OTU00832	Desulfovibrionales;Desulfovibrionaceae;unclassified_Desulfovibrionaceae	0	0.013043	0.001199
OTU01146	Clostridiales;Lachnospiraceae;unclassified_Lachnospiraceae	0.0075453	0.000296	0.001367
OTU01119	Clostridiales;Lachnospiraceae;unclassified_Lachnospiraceae	0.0003773	0.005336	0.001834
OTU00050	Bacteroidales;Porphyromonadaceae;Parabacteroides	0.1011065	0.450856	0.002202
OTU00186	Clostridia;Clostridiales;unclassified_Clostridiales	0.1844817	0.046538	0.00282
OTU00122	Clostridiales;Lachnospiraceae;Eisenbergiella	0.0211267	0.335252	0.002849
OTU00410	Clostridiales;Ruminococcaceae;Oscillibacter	0.0045272	0.034681	0.003348
OTU00058	Campylobacterales;Helicobacteraceae;Helicobacter	0.0580985	0.596103	0.003788
OTU00720	Clostridia;Clostridiales;unclassified_Clostridiales	0.0015091	0.01245	0.00456
OTU01984	Desulfovibrionales;Desulfovibrionaceae;unclassified_Desulfovibrionaceae	0	0.002668	0.006591
OTU01307	Clostridiales;Lachnospiraceae;unclassified_Lachnospiraceae	0.0003773	0.004743	0.007635
OTU01051	Bacteria;Firmicutes;unclassified_Firmicutes	0.0003773	0.007411	0.007741
OTU02591	Desulfovibrionales;Desulfovibrionaceae;unclassified_Desulfovibrionaceae	0	0.001779	0.008089
OTU01588	Bacteroidales;Porphyromonadaceae;Odoribacter	0.0033954	0.000296	0.008438
OTU00715	Desulfovibrionales;Desulfovibrionaceae;unclassified_Desulfovibrionaceae	0.0120724	0.003261	0.008709
OTU00460	Clostridiales;Lachnospiraceae;unclassified_Lachnospiraceae	0.0049044	0.049206	0.009747


### Bacterial Composition in Different Groups

**FIGURE 3:**
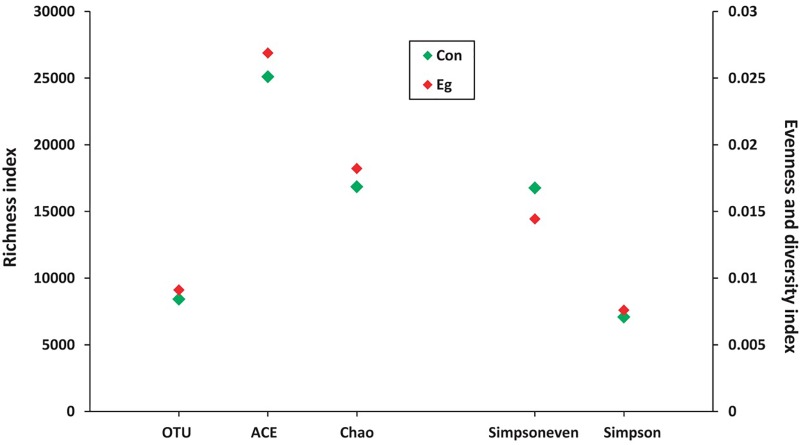
The microbial diversity variability in mice infected (Infected Group) or uninfected (Uninfected Group) with *E. granulosus*. LEfSe analysis showed the composition of the bacterial populations in the guts of the infected and uninfected mice was similar, whereas richness, evenness and diversity were no changed.

LEfSe analysis showed the composition of the bacterial populations in the guts of the infected and uninfected mice was similar, whereas richness, evenness and diversity were only slightly changed (Table 2 and Figure 3). In contrast, AMOVA analysis showed significant difference between the two groups for the microbiota (*P*_AMOV A_ = 0.029). Species richness (OTU, ACE and Chao index) was higher in the *E. granulosus* Infected Group, and the evenness (Shannoneven and Simpsoneven) was lower in this group compared with the uninfected mice. As richness and evenness combined, there was no significant difference in the diversity between the two groups (*p* > 0.5). Consequently, the results of these analyses indicated that *E. granulosus* infection did not alter much of the composition of the core bacteria present in the mouse gut significantly, although some rare bacteria in very low abundance were increased.

Among the 13 core families, LEfSe analysis showed no significant difference between the *E. granulosus* Infected Group and Uninfected Group in terms of microbiome. Among the major abundant genera, three showed significant differences between the groups (Table 4, LDA > 2, *p* < 0.05). The infected mice significantly increases two genera, including Eisenbergiella (1.9 times) and Parabacteroides (17.5 times) compared with the uninfected mice (*p* < 0.05).

### Predicted Functional Potential Changes in the Microbiomes of the *E. granulosus* Infection and Uninfected Groups

We used PICRUSt to predict and compare the microbial functional potential changes between the two groups. A total of 47 Kos were found to be significantly increased in the Infected Group (*p* < 0.05) (Table 6). At KEGG level 3, 7 pathways were identified as being significant difference (*p* < 0.05) between the Infected Group and Uninfected Group, and the changed pathways belonged to the metabolism category, including “Biotin metabolism,” “Biosynthesis and Biodegradation of secondary metabolites,” “Ether lipid metabolism,” “Steroid biosynthesis,” “Aminobenzoate degradation,” “Tryptophan metabolism” and “Limonene and pinene degradation.”

**Table 6:** Functional predictions using the PICRUSt base on 16S rRNA gene copy numbers.

KO accession	Annotation	Infected	Uninfected	*p*-value
K00655	1-acyl-sn-glycerol-3-phosphate acyltransferase [EC:2.3.1.51]	17,607	15,826	0.01887
K02312	2,3-dihydroxybenzoate-AMP ligase [EC:2.7.7.58]	4,214	3,074	2.43E-02
K01826	5-carboxymethyl-2-hydroxymuconate isomerase [EC:5.3.3.10]	2	0	0.015531
K01905	Acetyl-CoA synthetase (ADP-forming) [EC:6.2.1.13]	4,216	3,063	0.02436
K12554	Alanine adding enzyme [EC:2.3.2.-]	1	0	0.020538
K05341	Amylosucrase [EC:2.4.1.4]	4,223	3,068	0.023681
K03325	Arsenite transporter, ACR3 family	5,022	3,773	1.16E-02
K01338	ATP-dependent Lon protease [EC:3.4.21.53]	15,636	13,406	0.023016
K01305	Beta-aspartyl-dipeptidase (metallo-type) [EC:3.4.19.-]	4,245	3,082	0.023369
K01012	Biotin synthetase [EC:2.8.1.6]	15,209	12,941	0.013677
K03519	Carbon-monoxide dehydrogenase medium subunit [EC:1.2.99.2]	4,219	3,067	0.024608
K01647	Citrate synthase [EC:2.3.3.1]	14,507	12,412	0.023345
K01739	Cystathionine gamma-synthase [EC:2.5.1.48]	4,250	3,105	0.023501
K00901	Diacylglycerol kinase [EC:2.7.1.107]	5,333	4,032	0.012241
K11754	Dihydrofolate synthase/folylpolyglutamate synthase [EC:6.3.2.12 6.3.2.17]	26,899	24,234	0.011904
K07458	DNA mismatch endonuclease, patch repair protein [EC: 3.1.-.-]	4,883	3,692	0.01095
K06212	Formate transporter	4,268	3,116	0.021367
K13892	Glutathione transport system ATP-binding protein	4,226	3,072	0.024579
K04653	Hydrogenase expression/formation protein HypC	7,620	6,473	0.017468
K04654	Hydrogenase expression/formation protein HypD	7,624	6,475	0.016744
K04655	Hydrogenase expression/formation protein HypE	7,696	6,567	0.019062
K04656	Hydrogenase maturation protein HypF	7,630	6,479	0.016758
K04652	Hydrogenase nickel incorporation protein HypB	7,888	6,740	0.021211
K09384	Hypothetical protein	4,225	3,076	2.37E-02
K09703	Hypothetical protein	4,217	3,064	0.024347
K07301	Inner membrane protein	8,454	7,324	0.000592
K03779	L(+)-tartrate dehydratase alpha subunit [EC:4.2.1.32]	4,264	3,106	0.024966
K00879	L-fuculokinase [EC:2.7.1.51]	4,246	3,099	0.023788
K08369	MFS transporter, putative metabolite:H+ symporter	4,280	3,112	0.021188
K02018	Molybdate transport system permease protein	10,849	8,353	2.09E-02
K03637	Molybdenum cofactor biosynthesis protein C	5,564	4,311	0.024072
K07474	Phage terminase small subunit	4,318	3,190	0.022446
K02759	PTS system, cellobiose-specific IIA component [EC:2.7.1.69]	4,336	3,207	0.022898
K02760	PTS system, cellobiose-specific IIB component [EC:2.7.1.69]	4,448	3,372	0.019282
K02777	PTS system, glucose-specific IIA component [EC:2.7.1.69]	4,677	3,516	0.016117
K10026	Queuosine biosynthesis protein QueE	4,760	3,668	2.47E-02
K12996	Rhamnosyltransferase [EC:2.4.1.-]	4,219	3,080	0.024267
K03086	RNA polymerase primary sigma factor	17,918	16,008	3.41E-03
K05297	Rubredoxin-NAD+ reductase [EC:1.18.1.1]	4,352	3,216	0.020307
K03438	S-adenosyl-methyltransferase [EC:2.1.1.-]; 16S rRNA (cytosine1402-N4)-methyltransferase [EC:2.1.1.199]	16,924	15,121	0.014162
K07313	Serine/threonine protein phosphatase 1 [EC:3.1.3.16]	5,067	3,888	0.010963
K02945	Small subunit ribosomal protein S1	16,311	14,363	1.68E-03
K05814	sn-glycerol 3-phosphate transport system permease protein	4,248	3,118	0.023484
K11928	Sodium/proline symporter	4,419	3,257	0.022096
K01695	Tryptophan synthase alpha chain [EC:4.2.1.20]	9,531	8,500	0.021878
K07665	Two-component system, OmpR family, copper resistance phosphate regulon response regulator CusR	7,719	6,661	0.012516
K04784	Yersiniabactin non-ribosomal peptide synthetase	4,212	3,062	0.024426
Average		2,156	2,116	


*The numbers of Infected and Uninfected were the normalization of copy numbers. Uninfected, uninfected mice; Infected, mice infected with *E. granulosus*.*

### Correlations Between Bacterial Composition and Immunoglobulin Isotypes

**FIGURE 4:**
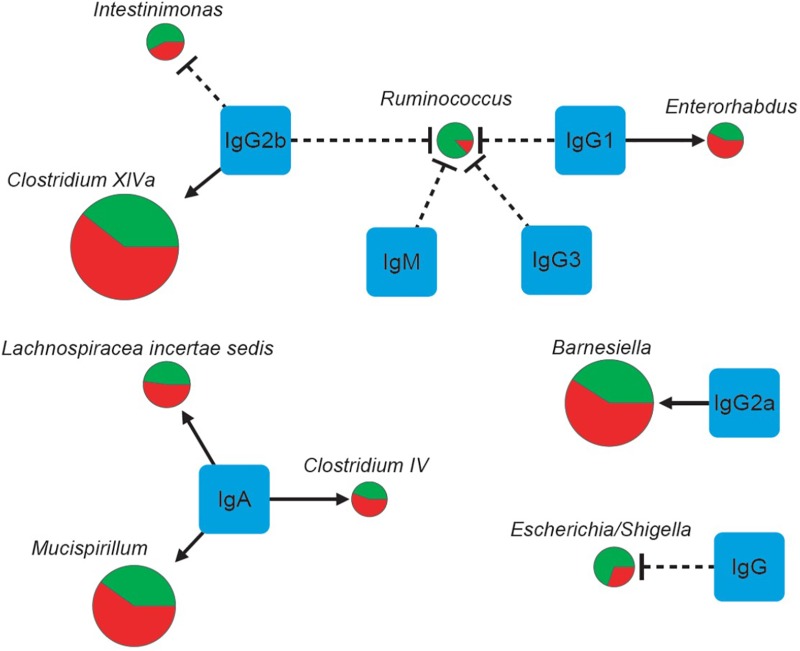
The correlations of seven physical factors and major genera. The pie charts show relative proportions of major genera in the *E. granulosus* infected mice and uninfected mice. (Green: Uninfected Group; red: Infection Group), and the circle size represents the reads numbers. Line and arrow type: solid arrow (positive relations), dash “T” (negative relations).

Spearman correlation analysis showed strong correlations of IgG, IgG1 and IgG2a with nine major genera (Table 7 and Figure 4). The numbers of *Enterorhabdus*, *Barnesiella* and *Clostridium* XlVa were positively correlated with IgG1, IgG2a and IgG2b levels, respectively. IgA was positively associated with increased numbers of genera *Clostridium* IV, *Lachnospiraceae Incertae sedis* and *Mucispirillum*. In addition, IgG, IgG1, IgG2b and IgG3 were associated with decreased numbers of genera *Escherichia/Shigella*, *Ruminococcus*, *Ruminococcus/Intestinimonas* and *Ruminococcus*, respectively (Table 7).

**Table 7:** Genera of bacteria correlating highly with serum antibody isotypes in *E. granulosus* infected mice and uninfected mice.

Genus	Uninfected (%)	Infected (%)	Factor	Uninfected	Infected	Spearman Coef	*p*-value
Barnesiella	2.06	2.99	IgG2a	0.2758	0.8682	0.9	0.037386
Ruminococcus	0.13	0.02	IgG1	0.2801	3.4034	–0.9	0.037386
Ruminococcus	0.13	0.02	IgG2b	0.2434	0.8148	–0.9	0.037386
Ruminococcus	0.13	0.02	IgG3	0.2329	1.0469	–1	0
Ruminococcus	0.13	0.02	IgM	0.3879	2.7058	–0.9	0.037386
Clostridium IV	0.04	0.05	IgA	0.1563	0.3758	0.9747	0.004818
Enterorhabdus	0.06	0.08	IgG1	0.2801	3.4034	0.9	0.037386
Intestinimonas	0.15	0.11	IgG2b	0.2434	0.8148	–0.9	0.037386
Escherichia/Shigella	0.39	0.17	IgG	0.7014	3.9271	–1	0
Lachnospiracea Incertaesedis	0.56	0.61	IgA	0.1564	0.3758	0.9747	0.004818
Mucispirillum	1.81	2.72	IgA	0.1564	0.3758	0.9747	0.004818
Clostridium XlVa	2.70	4.19	IgG2b	0.2434	0.8148	0.9	0.037386


*Uninfected, uninfected mice; Infected, mice infected with *E. granulosus*.*

## Discussion

The ecological balance of the microbiota in the gut is crucial for maintaining healthy condition (Cani et al., 2008). Disruption of the balance of the gut microbiota is associated with a range of diseases, including colorectal cancer, autoimmune diseases, metabolic diseases, among others (Sokol et al., 2008; Jiang et al., 2015). In this study, we showed that *E. granulosus* infection increased two genera of gut microbiota: *Eisenbergiella* and genus *Parabacteroides*, with most genera remaining unchange. The two genera are in the family *Lachnospiraceae*. Their increase may impact on human health (Plieskatt et al., 2013), and may be associated with diabetes in mice (Kameyama and Itoh, 2014).

At the OTU level, there were 150 OTUs significantly changed in the infected mice. However, among the OTUs, only 49 OTUs have taxonomic information at genus level with 101 without predicted taxonomic classification information, which limited our further analysis (Table 5). Additionally, the LEfSe analysis also showed genera *Eisenbergiella* and *Parabacteroides* increased in the infected mice, suggesting that these two genera of bacteria might be biomarkers for *E. granulosus* infection. In our study, the genus *Eisenbergiella* in the family *Lachnospiraceae* was up-regulated significantly in the infected mice, however, there is very limited biological function information on this genus. Combined with antibody analysis in this study, this genus of bacteria may be associated with a Th2 response.

Another increased genus in Infected Group is *Parabacteroides* whereas species belonging to the genus *Parabacteroides* are saccharolytic (Rajilic-Stojanovic and de Vos, 2014), being producers of short chain fatty acids (SCFAs) including acetate, propionate and butyrate as bacterial fermentation products (Cummings et al., 1987; Correa-Oliveira et al., 2016; Lloyd-Price et al., 2016). SCFAs act as links between the microbiota and the host immune system (Correa-Oliveira et al., 2016). The liver is the major systemic organ for SCFA metabolism and consumption (Kim et al., 2014), SCFAs released by the gut and equaled by hepatic uptake (Bloemen et al., 2009). *Parabacteroides* has evolved to contain a gene encoding a major capsid protein (Rosenwald et al., 2014) one of the phage orthologous groups (Kristensen et al., 2013). One report demonstrated that *Parabacteroides* was prevalent in diabetic (Wu et al., 2010). The increasing of genus *Parabacteroides* in hydatid infection may be associated with hepatic alteration.

Functional predictions showed seven pathways of the gut microbiota in the *E. granulosus* infection group were altered compared with the uninfected group. These pathways included biotin, lipid metabolism, and tryptophan metabolism. The synergistic effect of bacteria leads to the difference of gut flora metabolic pathways due to some or all intestinal bacteria involving in metabolism. Biotin metabolism in the intestine is regulated through transcriptional and post-transcriptional mechanisms. Its balance plays a key role in regulating the absorption and the function of biotin in tissues (Zoetendal et al., 2012). Based on pathway impact analysis, we found that tryptophan metabolism was decreased in the *E. granulosus* infection group. In mice infected with *schistosomes*, tryptophan or compounds from tryptophan metabolism were up-regulated and increased in urine which indicate possible problems in tryptophan metabolism in these infected animals (Njagi et al., 1992; Wang et al., 2004).

We showed that the bacterial composition of nine major genera had strong correlations with the levels of IgG, IgG1 and IgG2a antibodies against HCF antigens (Figure 4). The numbers of genera *Enterorhabdus*, and *Clostridium* XlVa were positively correlated with IgG1 and IgG2b levels, indicating that these bacteria can be tolerated with those Th2 associated antibodies or Th2 responses may benefit those genera of bacteria. Meanwhile, IgG2a, a Th1 associated antibody, was associated with increased number of genus *Barnesiella*, indicating Th1 has a role for increasing genus *Barnesiella*. Our data also showed that Th2 associated antibodies IgG1 and IgG2b and IgG3 decreased numbers of genera *Escherichia/Shigella*, and *Ruminococcus*, *Ruminococcus/Intestinimonas* (Table 7 and Figure 4). Interestingly, *Intestinimonas* decreased significantly in *E. granulosus* infection group as a differential genus by LEfSe analysis, perhaps it is associated with some kinds of change, then we concluded that it is highly related to IgG2b by Spearman coefficient correlation analysis and is consistent with the immune background of *E. granulosus* infection. So IgG2b may play impartment role in inhibition of *Intestinimonas*. *Clostridium* has been found to be associated with a number of diseases. It showed that *Clostridium* may participate in antibiotic-associated diarrhea (Buffie et al., 2015) and damages the human intestine *in vitro* (Fernandez Miyakawa et al., 2005). *Barnesiella* is present in the healthy intestinal tract and is influenced by antibiotics, and intestinal colonization with *Barnesiella* confers resistance to intestinal domination and bloodstream infection with vancomycin-resistant *Enterococcus* (Ubeda et al., 2013).

In summary, we explored gut microbiota in mice infected with *E. granulosus*, and found that chronic *E. granulosus* infection increased 101 OTUs including two genera of gut microbiota in mice. Functional prediction showed seven pathways of gut microbiota were altered, and bacterial composition of major genera had positive correlations with IgG1 and IgG2b in *E. granulosus* infected mice.

Whereas more than 85% of the genomic sequences between mouse and *Homo* are conserved, overall gene expression and its regulation are considerably different between the two species (Hugenholtz and de Vos, 2018). Human and mouse seems to be similar at phylum level, Bacteroidetes and Firmicutes are the two major bacterial phyla of the intestinal tract (Rawls et al., 2006). However, we do not know whether *E. granulosus* infection will affect human intestinal flora in the same way as the mouse and further studies are now required to understand the further possible mechanisms associated with altered colonization resistance after helminth infection and to determine changes in the gut microbiota of patients with CE.

## Accession Numbers

The sequence data have been submitted to the GeneBank Sequence Read Archive (Accession Number PRJNA396089).

## Author Contributions

WZ, JL, and DM contributed to conception and design of the study. JB organized the database. LH, WQ, and TW finished the animal experiments. HZ, ZZ, and YW performed the statistical analysis. JB and YW wrote the first draft of the manuscript. GG, XZ, and BG wrote sections of the manuscript. All authors contributed to manuscript revision, read and approved the submitted version.

## Conflict of Interest Statement

The authors declare that the research was conducted in the absence of any commercial or financial relationships that could be construed as a potential conflict of interest.
